# Sexual Dimorphism in the Association Between Neck Circumference and Handgrip Strength: A Population-Based Cross-sectional Study

**DOI:** 10.5152/eurasianjmed.2026.261507

**Published:** 2026-10-16

**Authors:** Idowu Elijah Adefisan, Inioluwa God’s gift Oretan

**Affiliations:** Department of Anatomy, University of Medical Sciences, Ondo, Nigeria

**Keywords:** Anthropometry, body composition, handgrip strength, muscle function, neck circumference, sex differences

## Abstract

**Background:**

Handgrip strength (HGS) is a well-validated proxy of overall muscle strength and nutritional status. Neck circumference (NC), an accessible and minimally invasive anthropometric measure, has emerged as a potential surrogate marker of upper-body composition. This study evaluated the predictive validity of NC for HGS in a mixed-sex population.

**Methods:**

This cross-sectional analysis included 450 participants (208 males, 242 females) aged 15-65 years. Anthropometric measurements included NC, HGS, height, weight, body mass index (BMI), and arm circumference. Bivariate associations were assessed using Pearson correlations, and multiple linear regression models were constructed to determine the independent contribution of NC to HGS, controlling for age, sex, BMI, height, and arm circumference. Sex-stratified analyses were conducted to examine differential effects.

**Results:**

Mean NC was 33.55 cm in males and 33.07 cm in females (*P* = .097). Handgrip strength was significantly higher in males (28.58 kg) than females (26.24 kg; *P* < .001). Overall, NC demonstrated a modest but significant correlation with HGS (*r* = 0.222, *P* < .001), though sex-stratified analyses revealed marked divergence: males showed a moderate correlation (*r* = 0.311, *P* < .001) while females showed none (*r* = 0.103, *P* = .109). In the fully adjusted model, NC independently predicted HGS (β = 0.372, 95% CI: 0.155 to 0.590, *P* = .001), contributing 2.26% unique variance. Sex-stratified regression confirmed NC as a significant predictor in males (β = 0.532, *P* < .001) but not females (β = 0.076, *P* = .643).

**Conclusion:**

Neck circumference independently predicts HGS in males but not females, suggesting sex-specific patterns in upper-body anthropometry and muscular function. Neck circumference may serve as a useful screening tool for muscle strength in male populations, particularly in resource-limited settings.

**Main Points**

- Neck circumference (NC) demonstrates significant predictive validity for handgrip strength (HGS) in males but not in females, indicating substantial sexual dimorphism in the relationship between upper-body anthropometry and muscular function.
- In male populations, NC may serve as a useful, field-deployable screening indicator of muscle strength, particularly in settings lacking sophisticated body composition assessment capabilities.
- Sex-stratified approaches are necessary in anthropometric research and clinical application when using NC as a proxy for musculoskeletal health.
- Neck circumference contributes approximately 2.3% unique explanatory variance beyond conventional anthropometric and demographic predictors of HGS.

## Introduction

Handgrip strength (HGS) has emerged as a robust, non-invasive biomarker of overall muscular function, nutritional status, and all-cause mortality risk across diverse clinical and population settings. As a reliable proxy for general muscle strength, HGS assessments have been integrated into geriatric evaluation protocols, sarcopenia screening algorithms, and nutritional surveillance systems worldwide.^1^^-^^4^ The predictive validity of HGS extends beyond musculoskeletal health to encompass cardiovascular disease, cognitive decline, and functional disability, establishing its utility as a comprehensive health indicator.^5^^,^^6^

Traditional determinants of HGS include demographic factors (age, sex), anthropometric measures (height, weight, and body mass index [BMI]), and lifestyle variables.^7^^,^^8^ However, the search for accessible, field-deployable anthropometric proxies of muscle function remains pertinent, particularly in low- and middle-income country (LMIC) settings where sophisticated body composition assessment techniques—such as dual-energy X-ray absorptiometry (DEXA) or bioelectrical impedance analysis (BIA)—are frequently unavailable.^9^^,^^10^

Neck circumference (NC) has garnered increasing attention as a simple, inexpensive, and reproducible anthropometric indicator with demonstrated associations with upper-body adipose distribution, lean mass, and metabolic risk factors.^11^^,^^12^ Anatomically, the neck region encompasses skeletal muscle structures (sternocleidomastoid, trapezius, and scalene muscles), adipose tissue, and vascular structures; imaging-based work has shown that paraspinal muscle area at the cervical spine correlates with whole-body skeletal muscle mass, supporting a direct mechanistic link between neck composition and overall musculoskeletal status.^13^Recent investigations have explored NC as a surrogate marker of obesity-related comorbidities, obstructive sleep apnea, and cardiovascular risk, though its relationship with muscular function remains inadequately characterized.^14^^,^^15^

The theoretical rationale for an NC–HGS association derives from the shared developmental and functional pathways governing upper-body musculature. Both NC and HGS reflect aspects of upper-limb skeletal muscle mass and function, potentially capturing common underlying physiological processes related to protein metabolism, hormonal status, and neuromuscular integrity.^16^ Furthermore, NC may serve as a composite indicator of both lean tissue and adipose components in the upper body, offering a holistic anthropometric assessment that transcends simple limb circumference measurements.^17^

Despite these theoretical considerations, empirical evidence regarding the NC–HGS relationship remains limited and inconsistent across populations. Existing studies have predominantly focused on elderly cohorts, clinical populations, or single-sex samples, restricting generalizability.^18^^,^^19^ Moreover, the potential moderating influence of sex on the NC–HGS association has received insufficient attention, despite well-established sexual dimorphisms in body composition, fat distribution patterns, and muscle mass distribution.^20^^,^^21^

The present study addresses these knowledge gaps by examining the predictive validity of NC for HGS in a population-based sample spanning a broad age spectrum (15-65 years) and including both sexes. The authors hypothesized that (1) NC would demonstrate a significant positive association with HGS independent of conventional covariates; (2) this association would be stronger in males than females due to sex-specific differences in upper-body muscle mass distribution; and (3) NC would contribute unique explanatory variance beyond established anthropometric predictors.

## Material and Methods

### Study Design and Participants

This cross-sectional study utilized data from 450 participants recruited through community-based sampling strategies between May 2025 and January 2026. The sample comprised 208 males (46.2%) and 242 females (53.8%), all of whom were residents of Ondo City, Ondo State, who self-identified as Yoruba, with both parents belonging to the same ethnic group up to the second filial generation. Participants ranged in age from 15 to 65 years (mean ± SD: 24.65 ± 8.26 years), and the broad age inclusion criteria were designed to capture developmental and age-related variations in body composition and muscular function. Prior to enrollment, all participants provided informed consent, and the study protocol received ethical approval from the Institutional Research Ethics Committee, University of Medical Sciences, Ondo, on April 18, 2025 (approval number: UNIMED-HREC/Apv/2025/172). This study did not use AI and/or LLM tools during the preparation of this manuscript.

### Anthropometric Measurements

#### Neck Circumference

Neck circumference was measured in centimeters using a non-stretchable tape positioned at the level of the laryngeal prominence (Adam’s apple), perpendicular to the long axis of the neck, with the participant standing erect and head positioned in the Frankfort horizontal plane. Measurements were taken at the end of normal expiration to standardize respiratory phase effects.

#### Handgrip Strength

Handgrip strength was assessed using a calibrated hand dynamometer following standardized procedures recommended by the American Society of Hand Therapists.^22^ Participants were seated with shoulders adducted and neutrally rotated, elbows flexed at 90°, and forearms in a neutral position. Three maximal effort trials were performed with the dominant hand, with 60-second rest intervals between attempts. The maximum value (kg) was recorded for analysis.

#### Additional Anthropometry

Height was measured to the nearest 0.1 cm using a stadiometer. Weight was measured to the nearest 0.1 kg using a calibrated digital scale. Body mass index was calculated as weight (kg)/height squared (m^2^). Arm circumference was measured at the mid-point between the acromion and olecranon processes. All measurements were performed by trained anthropometrists following standardized protocols. Figure 1 illustrates the anthropometric assessment procedure, including measurement of height, NC, arm circumference, weight, and HGS.

### Statistical Analysis

#### Descriptive Statistics

Continuous variables were summarized as mean ± SD. Normality was assessed using Shapiro–Wilk tests. Sex differences were evaluated using independent samples *t*-tests.

#### Bivariate Analyses

Pearson product–moment correlations were computed to examine zero-order associations between NC, HGS, and covariates. Spearman rank-order correlations were employed as non-parametric alternatives where distributional assumptions were violated.

#### Regression Analyses

Multiple linear regression models were constructed with HGS as the dependent variable. The primary model specification included NC as the predictor of interest, controlling for age, sex (dummy-coded), BMI, height, and arm circumference. Hierarchical regression was employed to quantify the unique variance contribution of NC (Δ*R*^2^) beyond the covariate set.

#### Sex-Stratified Analyses

Given the theoretical expectation of sexual dimorphism in body composition–muscle strength relationships, separate regression models were estimated for males and females. Model performance was compared using adjusted *R*^2^ and Akaike Information Criterion (AIC).

#### Diagnostic Analyses

Regression assumptions were evaluated through residual analysis, including examination of residual distributions (Q–Q plots), homoscedasticity (residuals vs. fitted plots), and influence statistics (Cook’s distance). Multicollinearity was assessed using variance inflation factors.

#### Software

All analyses were conducted using Python (Version 3.9; Python Software Foundation, Wilmington, DE, USA) with the statsmodels (v0.14), scipy (v1.11), and scikit-learn (v1.3) libraries. Statistical significance was set at *α* = 0.05 (2-tailed)

## Results

### Sample Characteristics

Table 1 presents descriptive statistics for the total sample and by sex. Males were significantly older, taller, and heavier than females (all *P *< .05). Body mass index was also modestly higher in males (*P* = .024).

Arm circumference did not differ significantly between sexes (*P* = .534).

Neck circumference did not differ significantly between sexes (*P* = .097), despite males demonstrating significantly greater HGS (*P* < .001).

### Bivariate Associations

Figure 2 presents a comprehensive analytical overview including scatterplots, correlation matrices, and regression diagnostics.

The overall correlation between NC and HGS was modest but statistically significant (*r* = 0.222, *P* < .001; *ρ* = 0.157, *P* < .001). However, sex-stratified analyses revealed marked divergence in these associations. In males, NC demonstrated a moderate positive correlation with HGS (*r* = 0.311, *P* < .001), whereas in females, the correlation was non-significant and substantially attenuated (*r* = 0.103, *P* = .109). This pattern persisted when using non-parametric Spearman correlations (males: *ρ* = 0.246, *P* < .001; females: *ρ* = 0.060, *P* = .352).

Correlation analyses across 3 age subgroups revealed that the NC-HGS association was significant in the 15-24 year group (*r* = 0.295, *P* < .001) and the 35-44 year group (*r* = 0.326, *P* = .011), with a non-significant association in the 25-34 year group (*r* = 0.046, *P* = .575).

### Multiple Regression Analyses

Table 2 presents results from the multiple linear regression model predicting HGS from NC and covariates. The full model explained 11.7% of variance in HGS (*R*^2^ = 0.117, adjusted *R*^2^ = 0.105; F(6443) = 9.75, *P* < .001).

Neck circumference emerged as a significant independent predictor of HGS (B = 0.372, 95% CI: 0.155-0.590, *P* = .001), with each centimeter increase in NC associated with approximately 0.37 kg greater grip strength, controlling for age, sex, BMI, height, and arm circumference. The standardized coefficient (β = 0.161) indicates that a 1 SD increase in NC corresponds to a 0.16 SD increase in HGS. Age also demonstrated a significant independent effect (B = 0.125, *P* = .002), consistent with developmental increases in muscle strength through early adulthood. Notably, sex, BMI, height, and arm circumference did not reach statistical significance as independent predictors when NC was included in the model.

Hierarchical regression was conducted to quantify the unique contribution of NC beyond established predictors, entering covariates (age, sex, BMI, height, and arm circumference) in Block 1 and NC in Block 2. The base model explained 9.4% of variance (*R*^2^ = 0.094, *P* < .001). Adding NC significantly improved model fit (Δ *R*^2^ = 0.023, F(1443) = 11.33, *P* < .001), indicating that NC contributes 2.3% unique explanatory variance beyond conventional anthropometric and demographic predictors.

### Sex-Stratified Regression Models

Table 3 presents the sex-stratified regression models. Given the divergent bivariate correlations, separate regression models were estimated for males and females.

#### Males

The model explained 14.0% of variance in HGS (adjusted *R*^2^ = 0.118). Neck circumference was the strongest predictor (B = 0.532, *P* < .001; β = 0.246), with each centimeter increase associated with 0.53 kg greater grip strength. No other covariates reached statistical significance, though BMI showed a marginal trend (*P* = .084).

#### Females

The model explained 10.0% of variance (adjusted *R*^2^ = 0.081). Critically, NC was not a significant predictor (B = 0.076, *P* = .643; β = 0.031). Instead, age (B = 0.186, *P *= .001) and height (B = 0.162, *P* = .005) emerged as significant predictors.

The male-specific model demonstrated superior explanatory power (higher *R*^2^, lower AIC) compared to the female model, and the NC coefficient in males was approximately 7 times larger than in females, confirming substantial sexual dimorphism in the NC–HGS relationship.

### Diagnostic Analyses

Figure 3 presents regression diagnostics and supplementary analyses including partial regression plots, predicted vs. observed values, and residual analyses.

Regression diagnostics indicated adequate model fit. Residual analysis revealed approximate normality (Q–Q plot), though slight heteroscedasticity was observed at higher fitted values. Variance inflation factors were all below 2.5, indicating acceptable multicollinearity levels. Cook’s distance identified no influential observations requiring exclusion.

## Discussion

This cross-sectional analysis of 450 participants provides novel evidence regarding the predictive validity of NC for HGS, with 3 principal findings. First, NC demonstrates a modest but significant independent association with HGS in the total population, contributing approximately 2.3% unique variance beyond conventional anthropometric and demographic predictors. Second, this association is driven entirely by a robust relationship in males (standardized β = 0.246, *P* < .001), with no significant association observed in females (β = 0.031, *P* = .643). Third, the male-specific regression model exhibits superior explanatory power compared to the female model, suggesting that NC captures sex-specific variation in upper-body composition relevant to muscular function.

The marked sexual dimorphism in the NC–HGS relationship represents the most salient finding of this investigation. Several mechanisms may account for this divergence. Males and females exhibit distinct patterns of fat distribution and muscle mass allocation. In males, NC predominantly reflects upper-body skeletal muscle mass (sternocleidomastoid, trapezius, and levator scapulae) and is less influenced by subcutaneous adipose deposition.^23^ Consequently, male NC may serve as a valid proxy for the musculoskeletal structures directly implicated in grip strength generation. In contrast, female NC may be more heavily influenced by subcutaneous adipose tissue, particularly in the submental and submandibular regions, potentially obscuring the relationship with underlying muscle function.^23^

Sex steroid hormones differentially regulate muscle mass distribution and adipose tissue patterning. Testosterone promotes upper-body muscle hypertrophy and visceral fat accumulation, while estrogen favors gluteofemoral fat deposition.^24^^,^^25^ These hormonal effects may render NC a more valid indicator of muscle mass in males compared to females.

Grip strength itself demonstrates sexual dimorphism in its determinants. In a sample of university-aged men and women, HGS correlated strongly with fat-free mass and skeletal muscle mass in men, whereas these associations were weak and non-significant in women, whose grip strength instead tracked more closely with stature.^26^ The authors’ finding that height (a proxy for overall body size) predicts HGS in females but not males supports this interpretation.

Previous investigations linking anthropometric indicators to HGS have yielded inconsistent results across populations, likely reflecting heterogeneous sample compositions and analytical approaches. In a population-based sample of 1500 Portuguese older adults, lower mid-arm muscle circumference was associated with reduced HGS, but only in men, not women, echoing the male-specific pattern observed in the present study.^27^ Literature specifically linking NC to HGS remains comparatively sparse, and sex-stratified normative approaches to grip strength, such as T-score-based classification of dynapenia, have themselves been developed and validated separately by sex, underscoring the broader recognition that HGS determinants and reference standards should not be assumed to generalize across sexes.^28^ The authors’ findings, showing a significant NC–HGS association restricted to males, align with this pattern of sex-specific musculoskeletal relationships and reinforce the necessity of sex-stratified analyses for valid inference.

The magnitude of the male-specific association (*r* = 0.31, β = 0.25) is comparable in size to correlations reported between HGS and other upper-body anthropometric measures, such as arm circumference (*r* = 0.15, in the data), and is broadly consistent with HGS’s established role as a robust, sex-relevant indicator of muscle-related outcomes across the adult lifespan.^29^ This study found that the U-shaped pattern across the lifespan may reflect developmental consolidation of muscle mass in early adulthood and accelerated sarcopenic changes in later years. Together, these findings suggest that NC offers predictive validity comparable to or exceeding established anthropometric indicators, at least in male populations.

The present findings carry several practical implications for clinical and community-based health assessment. Given that NC measurement requires nothing more than a flexible tape measure and minimal technical training, it emerges as a highly feasible screening modality, particularly in resource-constrained settings where sophisticated anthropometric equipment may be unavailable. Importantly, however, the sex-specific nature of the NC and HGS relationship underscores the necessity of adopting sex-stratified reference standards and interpretation frameworks, as applying uniform cut-offs across sexes risks misclassification and undermines the clinical utility of the measure. In male populations specifically, NC shows particular promise as a frontline screening tool for identifying individuals at risk of low muscle strength, a finding with meaningful downstream implications given that such identification could prompt timely referral for further nutritional or functional assessment before deficits become clinically significant.

However, the modest effect sizes (*R*^2^ = 0.14 in males) indicate that NC alone is insufficient for precise HGS prediction. Rather, NC should be conceptualized as 1 component of a multifactorial assessment battery including age, body size measures, and functional evaluations.

### Limitations

Strengths of this study include the population-based sampling strategy, broad age range, balanced sex distribution, and rigorous anthropometric protocols. The explicit examination of sex differences and the use of hierarchical regression to quantify unique variance contributions represent methodological advances over prior correlational analyses.

Limitations warrant consideration. The cross-sectional design precludes causal inference regarding developmental trajectories or intervention effects. The sample was drawn from a specific geographic and cultural context, potentially limiting generalizability to other populations. The authors did not assess inter-rater reliability of anthropometric measurements, though standardized protocols were employed. Finally, the absence of direct body composition assessment (e.g., DEXA) precludes definitive determination of whether the observed sex differences reflect differential fat–muscle composition of the neck region.

### Future Research Directions

Several avenues for future inquiry emerge from these findings. Longitudinal studies should examine whether NC trajectories predict changes in HGS over time, particularly during periods of rapid muscle accrual (adolescence) or loss (aging). Intervention studies could assess whether resistance training-induced changes in NC correlate with HGS improvements. Mechanistic investigations incorporating imaging techniques (ultrasound, magnetic resonance imaging) are needed to elucidate the tissue composition of the neck region and its relationship to upper-limb muscle function across sexes.

## Conclusion

Neck circumference demonstrates significant predictive validity for HGS in males but not in females, indicating substantial sexual dimorphism in the relationship between upper-body anthropometry and muscular function. In male populations, NC may serve as a useful, field-deployable screening indicator of muscle strength, particularly in settings lacking sophisticated body composition assessment capabilities. These findings underscore the necessity of sex-stratified approaches in anthropometric research and clinical application, and contribute to the emerging evidence base regarding accessible proxies of musculoskeletal health.

## Figures and Tables

**Figure 1.**
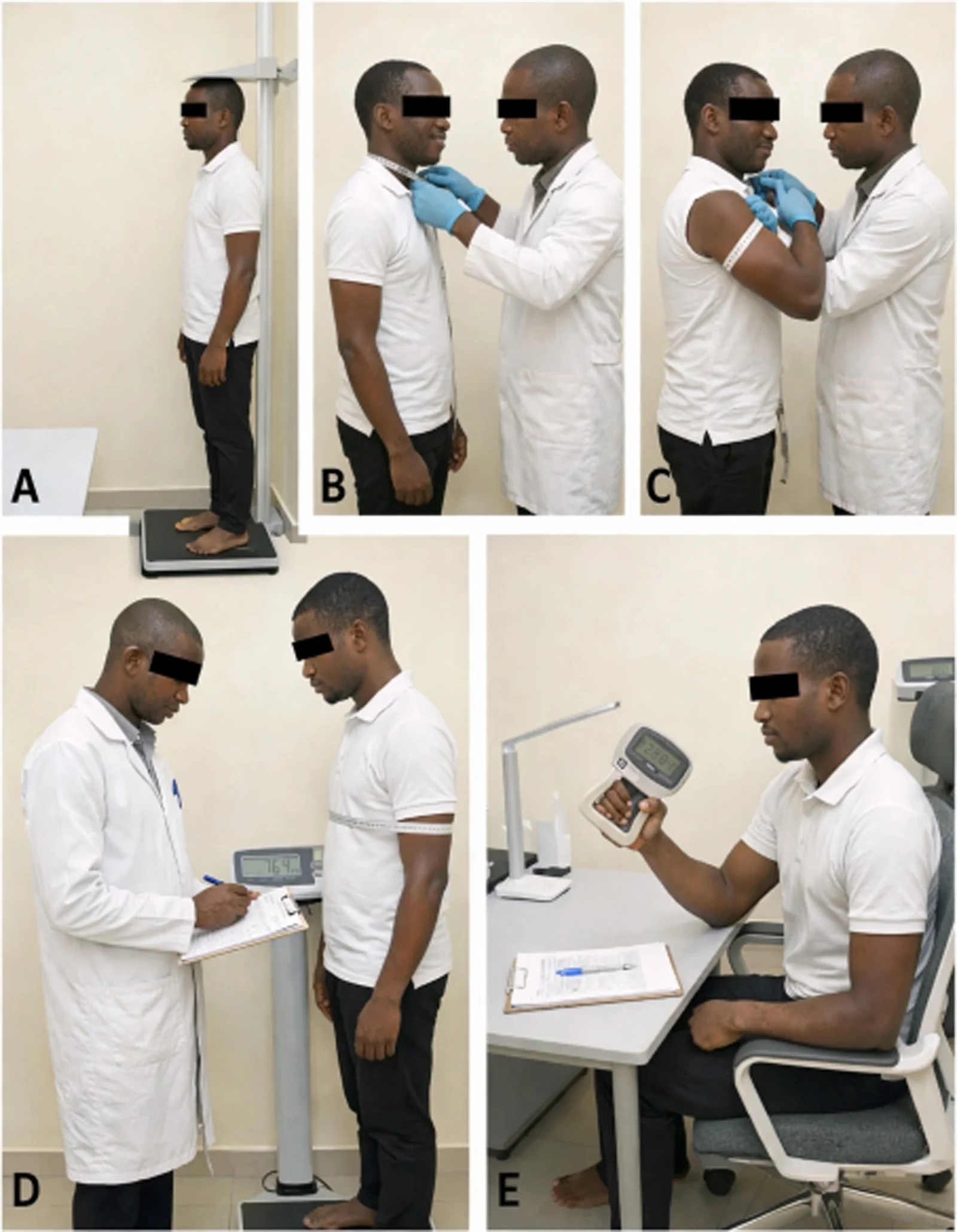
Procedure for anthropometric assessment: (A) height, (B) neck circumference, (C) arm circumference, (D) weight measurement and data recording, and (E) handgrip strength assessment.

**Figure 2.**
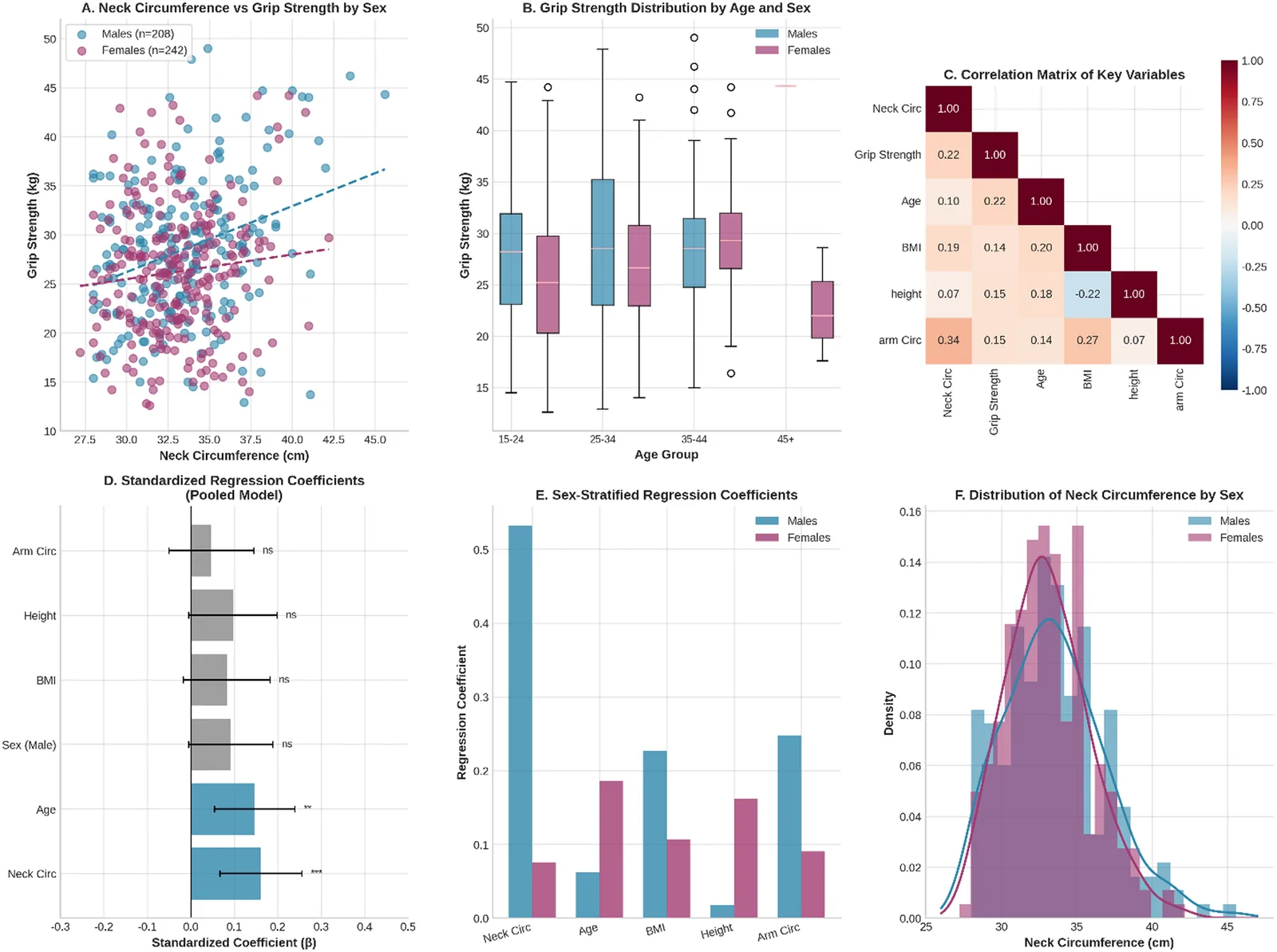
Comprehensive analytical overview. (A) Scatterplot of neck circumference vs. grip strength by sex with fitted regression lines; (B) Box plots of handgrip strength distribution across age groups and sex; (C) Correlation matrix heatmap of key anthropometric variables; (D) Standardized regression coefficients (β weights) from the pooled multiple regression model with 95% CIs; (E) Sex-stratified unstandardized regression coefficients; (F) Distribution density of neck circumference by sex.

**Figure 3.**
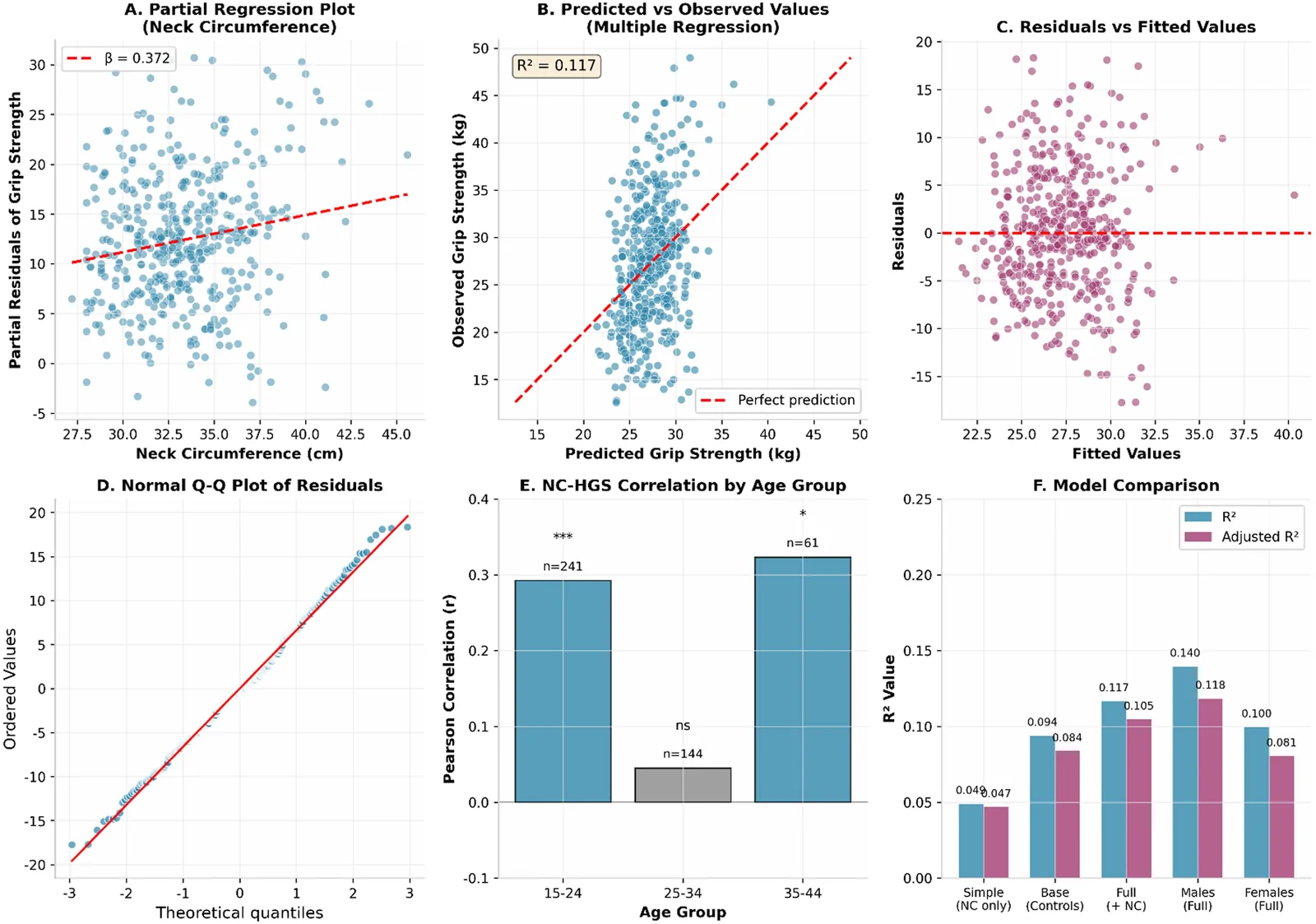
Regression diagnostics and supplementary analyses. (A) Partial regression plot for neck circumference; (B) Predicted vs. observed grip strength values; (C) Residuals vs. fitted values; (D) Normal Q–Q plot of residuals; (E) Age-stratified NC–HGS correlations; (F) Model comparison across specifications.

**Table 1.** Descriptive Statistics by Sex

Variable	Overall (n = 450)	Males (n = 208)	Females (n = 242)	*P*
Age (years)	24.65 ± 8.26	25.80 ± 8.50	23.67 ± 7.94	.006*
Height (cm)	167.62 ± 9.29	171.18 ± 9.58	164.56 ± 7.85	<.001***
Weight (kg)	57.52 ± 11.56	61.33 ± 11.66	54.24 ± 10.43	<.001***
BMI (kg/m squared)	20.51 ± 4.06	20.98 ± 4.00	20.11 ± 4.09	.024*
Neck circumference (cm)	33.29 ± 3.04	33.55 ± 3.33	33.07 ± 2.77	.097
Arm circumference (cm)	26.35 ± 3.34	26.25 ± 3.20	26.44 ± 3.47	.534
Hand grip strength (kg)	27.32 ± 7.02	28.58 ± 7.19	26.24 ± 6.71	<.001***

Values are mean ± SD. *P*-values from independent samples *t*-tests.

*P < .05

**P < .01

***P < .001

**Table 2.** Multiple Linear Regression Predicting Handgrip Strength

Variable	B	SE	β	*t*	*P*	95% CI
Intercept	−6.628	7.341	—	−0.903	.367	(-−21.06, 7.80)
Neck circumference	0.372	0.111	0.161	3.366	.001**	(0.16, 0.59)
Age	0.125	0.040	0.147	3.132	.002**	(0.05, 0.20)
Sex (Male)	1.300	0.694	0.092	1.874	.062	(-−0.06, 2.66)
BMI	0.144	0.088	0.083	1.640	.102	(-−0.03, 0.32)
Height	0.073	0.039	0.097	1.880	.061	(-−0.00, 0.15)
Arm circumference	0.100	0.104	0.048	0.960	.337	(-−0.10, 0.30)

*R*^2^ = 0.117, adjusted *R*^2^ = 0.105, F(6443) = 9.75, *P* < .001, AIC = 2988.74.

β, standardized coefficient; B, unstandardized coefficient; SE, standard error.

*P < .05.

**P < .01.

***P < .001.

**Table 3.** Sex-Stratified Multiple Regression Models

Variable	B (M)	*P* (M)	β (M)	B (F)	*P* (F)	β (F)
Intercept	-5.209	.622	—	-11.787	.265	—
Neck circumference	0.532	<.001***	0.246	0.076	.643	0.031
Age	0.062	.306	0.073	0.186	.001**	0.220
BMI	0.227	.084	0.126	0.107	.366	0.065
Height	0.018	.740	0.024	0.162	.005**	0.189
Arm Circumference	0.248	.126	0.110	0.091	.512	0.047

Model fit for males: *R*^2^ = 0.14, adjusted *R*^2^ = 0.118, AIC = 1390.78. Model fit for females: *R*^2^ = 0.10, adjusted *R*^2^ = 0.081, AIC = 1593.42.

M, males (n = 208); F, females (n = 242).

*P < .05.

**P < .01.

***P < .001.

## Data Availability

The data that support the findings of this study are available on request from the corresponding author.
